# Amygdala Low-Frequency Stimulation Reduces Pathological Phase-Amplitude Coupling in the Pilocarpine Model of Epilepsy

**DOI:** 10.3390/brainsci10110856

**Published:** 2020-11-13

**Authors:** István Mihály, Károly Orbán-Kis, Zsolt Gáll, Ádám-József Berki, Réka-Barbara Bod, Tibor Szilágyi

**Affiliations:** 1 Department of Physiology, Faculty of Medicine, George Emil Palade University of Medicine, Pharmacy, Science, and Technology of Târgu Mureș, 540142 Târgu Mureș, Romania; karoly.orban-kis@umfst.ro (K.O.-K.); berki.adam@yahoo.com (Á.-J.B.); bod.reka@yahoo.com (R.-B.B.), tibor.szilagyi@umfst.ro (T.S.); 2 Department of Pharmacology and Clinical Pharmacy, George Emil Palade University of Medicine, Pharmacy, Science, and Technology of Târgu Mureș, 540142 Târgu Mureș, Romania; zsolt.gall@umfst.ro

**Keywords:** temporal lobe epilepsy, pilocarpine, amygdala, deep brain stimulation, hippocampus, phase-amplitude coupling, interictal discharges

## Abstract

Temporal-lobe epilepsy (TLE) is the most common type of drug-resistant epilepsy and warrants the development of new therapies, such as deep-brain stimulation (DBS). DBS was applied to different brain regions for patients with epilepsy; however, the mechanisms of action are not fully understood. Therefore, we tried to characterize the effect of amygdala DBS on hippocampal electrical activity in the lithium-pilocarpine model in male Wistar rats. After status epilepticus (SE) induction, seizure patterns were determined based on continuous video recordings. Recording electrodes were inserted in the left and right hippocampus and a stimulating electrode in the left basolateral amygdala of both Pilo and age-matched control rats 10 weeks after SE. Daily stimulation protocol consisted of 4 × 50 s stimulation trains (4-Hz, regular interpulse interval) for 10 days. The hippocampal electroencephalogram was analyzed offline: interictal epileptiform discharge (IED) frequency, spectral analysis, and phase-amplitude coupling (PAC) between delta band and higher frequencies were measured. We found that the seizure rate and duration decreased (by 23% and 26.5%) and the decrease in seizure rate correlated negatively with the IED frequency. PAC was elevated in epileptic animals and DBS reduced the pathologically increased PAC and increased the average theta power (25.9% ± 1.1 vs. 30.3% ± 1.1; *p* < 0.01). Increasing theta power and reducing the PAC could be two possible mechanisms by which DBS may exhibit its antiepileptic effect in TLE; moreover, they could be used to monitor effectiveness of stimulation.

## 1. Introduction

Temporal lobe epilepsy (TLE) is one of the most common chronic neurological disorder and it severely affects the person’s quality of life [1]. Cellular and network level hyperexcitability is present in recurrent seizures and histopathological changes that involve temporal lobe structures like the hippocampus and amygdala [2]. Almost one-third of the patients diagnosed with TLE remain resistant to the classical pharmacological treatment [3,4]; moreover, the ablative surgery procedures are not applicable in many of these cases [5,6]. Therefore the development of alternative therapeutic approaches, such as deep brain stimulation (DBS), received increasing interest in the last decades [7,8,9]. Despite DBS’s more and more widespread usage in different neurological illnesses [10] as well as for medically refractory TLE [11,12], its mechanisms of action are not fully elucidated [13,14], and there is a substantial variability regarding the epileptic patients’ response to treatment [12,15,16].

The amygdala plays an important role in temporal lobe epilepsy [17,18], and its electrical stimulation with low frequency (LFS, e.g., with 1-4 Hz) can decrease the epileptiform electrical activity if at least four packages with at least 200 pulses are administered with 5 min pauses between the stimuli [19,20]. High frequency stimulation (HFS) was demonstrated to be more effective in reducing seizures and is often used in DBS therapies [21,22], but LFS also showed antiepileptic effect in a couple of human trials [23,24] and in several animal models of epilepsy [25,26,27,28]. LFS may be associated with reduced target tissue damage due to the smaller amount of stimulating current [29,30]. The underlying mechanisms of HFS vs. LFS induced seizure suppression are most likely different. LFS probably reduces the occurrence of spontaneous excitatory postsynaptic currents, increasing inhibitory postsynaptic currents, and restoring glutamatergic and GABAergic transmission to normal [31,32]. Moreover, it increases the seizure threshold and can reduce seizure numbers in pilocarpine and pentylenetetrazole (PTZ) models of epilepsy, even if it is applied unilaterally [25,28].

As well-established and widely used stimulation protocols are still lacking, it is important not only to characterize the underlying physiological mechanisms but to comprehensively evaluate the stimulation and to find biomarkers revealing its effectiveness [33]. Electrophysiological recordings can provide important information regarding seizure type and onset zone, but can also be used to detect interictal epileptiform discharges (IEDs), which were shown to occur more frequently in epileptic patients [34]. Moreover, spectral analysis of the electroencephalogram (EEG) signal shows decreased theta band power in TLE [35], and its increase during therapy may reflect an antiepileptic action [36]. Another tool to measure the network excitability is the analysis of cross-frequency couplings [37]. The phase-amplitude coupling (PAC) between low-frequency bands (e.g., delta) and higher frequencies is known to be increased during and before epileptic seizures [38,39]. Recently it was proposed to optimize neuromodulation techniques based on monitoring PAC in various neurologic disorders [40].

In the present study, we investigated the effect of amygdala low-frequency stimulation in the lithium-pilocarpine model of TLE in rats during the early chronic phase of the disease. We aimed to observe the electrophysiological changes that appear in the hippocampus during deep brain stimulation. We hypothesize that by increasing the relative theta power and by weakening the dependence of high frequencies’ amplitude on slow frequencies’ phase, DBS could alleviate the increased synchronicity between neural networks in the epileptic tissue, and these mechanisms could be key operating principles of DBS.

## 2. Materials and Methods

### 2.1. Animals

All experiments were conducted in accordance with the 2010/63/EU directive of the European Parliament and the local regulations approved by the Ethics Committee for Scientific Research of the George Emil Palade University of Medicine, Pharmacy, Science, and Technology of Târgu Mureș (ethical committee license no: 340/17 November 2017, extended by no. 54/2 April 2019). The animals used in the study were male Wistar rats (~100 g) aged 6 weeks at the time of the induction of status epilepticus (SE), as survival rate is the highest at this age [41,42]. They were housed in standard plexiglas cages under controlled environmental (~23 °C) and light conditions, i.e., 12 h dark-light cycle, with ad libitum access to food and water. The animals were randomly divided into three groups: stimulated epileptic (DBS-Pilo, *n* = 10), stimulated healthy control (DBS-Control, *n* = 4), and non-stimulated healthy control (SHAM-Control, *n* = 3); therefore, only the DBS-Pilo group underwent the induction.

### 2.2. Status Epilepticus Induction and Video Monitoring

We used the lithium-pilocarpine model of temporal lobe epilepsy, as it reproduces almost all aspects of human TLE—initial injury, latent period, increasingly more severe and more frequent seizures [43,44]. At 16–22 h before pilocarpine administration, lithium chloride was injected intraperitoneally (i.p.) (127 mg/kg, Sigma-Aldrich, St. Louis, MO, USA) to potentiate the effect of pilocarpine and reduce the necessary dose of pilocarpine. At 20 min before pilocarpine injection, methylscopolamine (1 mg/kg, Sigma-Aldrich, St. Louis, MO, USA) was given. Pilocarpine-hydrochloride (30 mg/kg, Sigma-Aldrich, St. Louis, MO, USA) was administered i.p., under continuous video monitoring. SE was defined as a continuous seizure that lasted longer than 30 min. Seizures were classified according to the revised Racine scale [45], and only rats having Racine 5 and Racine 6 grade seizures were included in the study. SE was stopped after 120 min from the pilocarpine administration, with diazepam (5 mg/kg, i.p. Terapia-Ranbaxy, Cluj-Napoca, Romania). Animals recovered from SE in approximately 2–3 h. Diazepam doses were repeated if convulsive behavior persisted. After i.p. rehydration each animal was placed separately in a standard plexiglas observation box. Control animals received only lithium and i.p. saline solution, and a single dose of diazepam. They were kept in similar housing conditions and diet as DBS-Pilo rats. Each rat was continuously video-monitored (24/24 h, 7 days/week) throughout the whole study. Data were analyzed offline by manually going through the whole recording to detect spontaneous behavioral seizures and behavioral abnormalities. Observers were not blind to the experimental conditions. The exhibited seizures were categorized according to the revised Racine scale [45], and only grade 3 to 6 seizures were quantified (See Supplementary File, Section S1).

### 2.3. Stereotaxic Procedures

Electrode implantation was performed stereotaxically 7–8 weeks after SE. Anesthesia was induced by isoflurane (5%) followed by i.m. injection of a mixture of ketamine (100 mg/kg LeVet. Beheer B.V., Oudewater, The Netherlands) and xylazine (10 mg/kg, Bioveta, Ivanovice na Hané, Chech Republic). All electrodes and their connectors were purchased from Plastics One Inc. (Roanoke, VA, USA) and were implanted according to The Rat Brain atlas of Paxinos and Watson [46], with the bregma suture as reference. Hippocampal recording electrodes were two Polyimide coated 0.2 mm diameter single-stranded stainless-steel monopolar electrodes implanted bilaterally (AP: −3.6 mm, ML: ± 2.6 mm, DV: 3.6 mm to skull surface). The stimulation electrode consisted of a bipolar polyimide coated twisted stainless-steel electrode (diameter: 0.2 mm, tip distance: 0.125 mm) which was implanted in the left basolateral amygdala (BLA, AP: −2.8 mm; ML +5 mm to bregma; DV +8.4 mm). The distance of the stimulation electrode (always left side) to the left hippocampal recording electrode was 5.4 mm, while to the right hippocampal recording electrode it was 9 mm (See Supplementary File, Section S3). The recording electrodes were connected to a 6-channel plastic connector, the stimulation electrode to a 2-channel connector, and both were fixed to the skull with dental acrylic cement (Duracryl, Spofa Dental, Markova, Czech Republic). Correct electrode positions were verified by Nissl staining, at the end of the experiment. Four stainless-steel Teflon coated screw electrodes were drilled to the depth of dura. Two of them were used as epidural recording electrodes (AP: +2 mm, ML: ±2 mm) and two were placed at the posterolateral part of the parietal bones and used as reference and ground electrodes. At the end of the procedure, a local anti-inflammatory and antibiotic (Tobramycin–Dexamethasone) solution was applied on the site of intervention.

### 2.4. EEG/Local Field Potential (LFP) Recording

Following a 10-day long post-surgical recovery, animals were kept in custom-built plexiglas cages (40 × 45 × 50 cm) housed in a Faraday shielded enclosure, with food and water ad libitum and a 12 h dark/light cycle (light on at 7 a.m.). The 6-channel and 2-channel plastic connectors were connected to a swivel contact via corresponding recording and stimulation cables, which made it possible to record EEG and to apply electrical stimulation at the same time on a freely moving rat. The electrical activity was recorded via an 8-channel preamplifier and amplifier system (SUPERTECH Multiamp SMA-4a, Supertech, Pécs, Hungary). The amplified signal was passed through on a 0.16 Hz low-pass filter, 8 kHz high-pass filter, and a 50 Hz Notch filter. Data acquisition was done by an A/D converter (PCI 6036E, National Instruments, Austin, TX, USA) with a sampling rate of 5 kHz (See Supplementary File, Section S2).

### 2.5. Stimulation Protocol

Stimulation was carried out by using a BioStim gate-controller and pulse pattern generator (STC-8b) connected to a Bipolar Floating End-stage (BSE-3b; Supertech, Pécs, Hungary). The morphology of the stimulation signal was verified by a shunt resistor connected to an oscilloscope before and at the end of the experiments.

**Figure 1:**
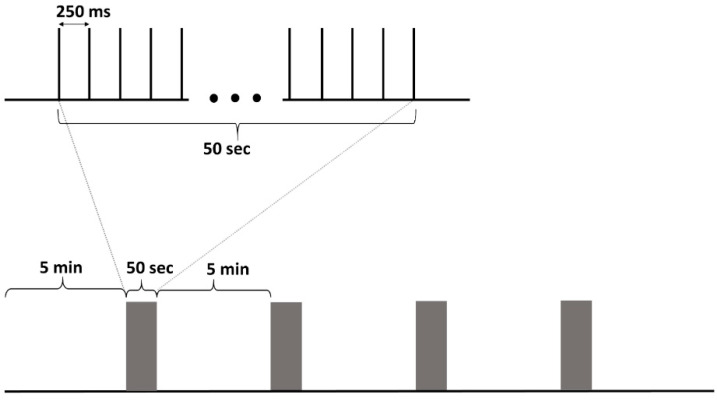
The stimulation protocol. A stimulation train consisted of 50 s, 4 Hz, continuous, regular interstimulus interval stimulation. The daily stimulation package consisted of 4 stimulation trains separated by 5 min pauses. 5-min pre- and post-stimulation period was recorded too.

Biphasic, square pulses with 100 µs duration were used. The inter-pulse interval was regular, at 4 Hz. The current amplitude was determined on the first day of the experiment by starting at 100 µA, and then it was increased gradually in steps of 100 µA until a maximum of 500 µA. If behavioral changes (like twitching, blinking, freezing or motionless stare) or epileptiform EEG were not elicited a 500 µA amplitude was used during the whole period, otherwise, it was reduced by 100 µA. Stimulation was performed for 10 days (daily, between 3 p.m. and 4 p.m.), a daily package consisted of a 4 × 50 s train with 5 min pause between each train. EEG recording was started 5 min before the first train and ended 5 min after the last (Figure 1).

### 2.6. Histology

At the end of the 10 days-long stimulation protocol, rats were deeply anesthetized with a mixture of Ketamine-Xylazine (90 mg/kg + 10 mg /kg) and transcardially perfused with ice-cold normal saline solution (0.9%, 1 min 30 s) followed by 4% paraformaldehyde in 0.1 M phosphate buffer (pH 7.4) solution containing 15% picric acid. A total of 60 µm coronal sections were cut by using a Leica vibratome (Leica VT 1000S, Leica, Germany). Electrode positions were checked visually during slicing and confirmed by light microscopy after cresyl-violet staining.

### 2.7. Interictal Discharges Analysis 

Hippocampal local field potential was recorded before, during, and after the electrical stimulation of the amygdala. EEG recordings were analyzed offline with Spike 2 software package (Cambridge Electronic Design, Cambridge, UK). Interictal epileptiform discharges were identified manually on the whole recording. They were counted in both left and right hippocampal recordings and were averaged. The following interictal discharges were documented: spikes, polyspikes, and sharp waves, as defined by others [34,47], if their amplitude was greater than 2 SD of background activity.

### 2.8. Spectral Analysis

EEG recordings were converted to European Data Format. Fast Fourier Transform (FFT) was performed on hippocampal LFP epochs using the EDF browser (Teunis van Beelen, The Netherlands). 10-s sliding windows with a 50% overlap were chosen to perform FFT. Based on electrophysiological considerations, delta (1–4 Hz), theta (4–12 Hz), beta (15–30 Hz), and gamma-fast ripple (30–600 Hz) frequency bands were distinguished. Relative theta power was calculated and averaged. To evaluate short-term effects evoked by DBS on LFP theta power of the immediate prestimulation and poststimulation 30 s time intervals were compared.

### 2.9. Phase-Amplitude Coupling (PAC)

From each daily 30 min long EEG recording, the pre-stimulation 5-min intervals and the last 5-min intervals (after the last stimulation train) were selected for analysis both in the case of DBS-Pilo and DBS-Control groups. For the SHAM-Control animals’ 30-min recordings, the first and last 5-min intervals were extracted and analyzed.

The frequency for phase (fP) was represented by delta oscillations (1–4 Hz), the frequency for amplitude (fA) consisted of frequency ranges 30–100 Hz for gamma, 100–150 Hz for HFOs, 150–250 Hz for ripples, and 250–600 Hz for fast ripples. To estimate the PAC, a time-resolved phase-amplitude coupling measure (tPAC) was applied by using the Brainstorm software. We calculated tPAC separately between the delta and each aforementioned high-frequency range, so on each 5-min long recording, four different calculation were performed [48]. tPAC is a novel method to obtain a PAC comodulogram and it detects the peak value of coupling strength between slow and fast rhythms over sliding time windows along with the selected recordings. It has the advantage that it detects the coupling strength with high sensitivity and high immunity to noise. It performs better than other existing methods, when using short data length [49].

Considering the length of our epochs (5 min) and the given ranges for fP (1–4 Hz), to optimize the process we used 18-s long time windows, with a 50% overlap between them [48].

### 2.10. Statistical Analysis

Data analysis was performed using statistical software GraphPad PRISM 8. One-way analysis of variance (ANOVA), paired t or unpaired *t*-tests were used to detect possible differences between groups, if not stated otherwise. Data are presented as mean ± S.E.M. For all analyses, an alpha value < 0.05 was considered significant.

## 3. Results

### 3.1. Seizure Rate and Duration

**Figure 2:**
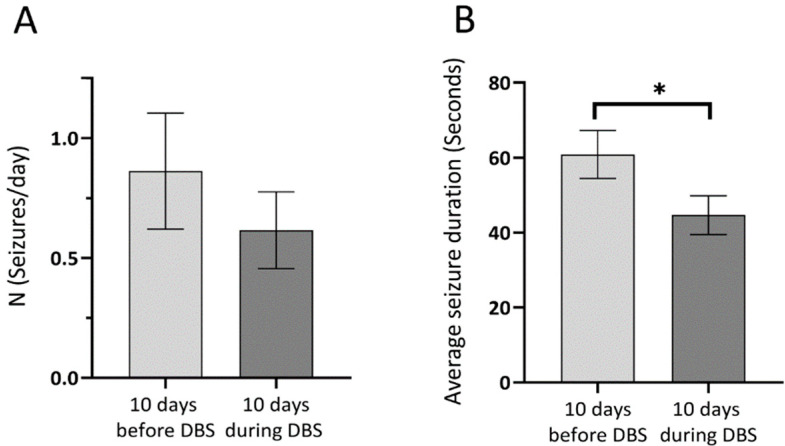
The effect of deep brain stimulation (DBS) on seizure rate and duration during the early chronic phase of the epilepsy, before and during the stimulation. (**A**) Seizure rate (seizures/day) 10 days before and during the 10 days DBS period (*n* = 10). A 23% decrease in seizure frequency was present. (**B**) Average seizure duration 10 days before and 10 days during DBS. A significant reduction in seizure duration can be observed during DBS. Data are presented as mean ± SEM, * *p* < 0.05.

All rats receiving pilocarpine had spontaneous recurrent seizures (*n* = 14/14). Mortality was 21% amongst inducted animals. One animal which survived the SE developed torticollis and could not be included in the study. The seizure-free (latent) period lasted 14–21 days, being followed by the chronic period. Only Racine 3–6 stage seizures were identified based on video recordings. The SHAM-Control and DBS-Control animals had no behavioral seizures. A progressive increase in seizure number was observed during the chronic period in pilocarpine animals (*n* = 10). The electrode implantation had no significant impact on seizure numbers, as the seizure rates one week before and one week after the surgical procedure were 0.72 vs. 0.74 seizures/day. Comparing the 10-day periods before and during DBS, an overall 23% decrease in seizure number was noticed (Figure 2A). Out of the 10 animals, 6 had a decrease in seizure rate and 4 had the same seizure rate.

To evaluate the short-term effect of DBS on seizure numbers, the 4-h periods before and after stimulation packages were compared, showing a 30% decrease in seizure rate (1.18 seizure/day vs. 0.83 seizure/day). The average duration of seizures based on video recordings before DBS was 60.84 s, while during the 10 days of DBS it was 44.67 s, a significant reduction by 26.5% (Figure 2B). The highest reduction was in animals that had longer mean seizure durations before DBS, and seizure duration reduction correlated significantly with the length of initial seizure durations (r = 0.934, *p* < 0.001). 

### 3.2. Interictal Epileptiform Discharges

Hippocampal EEG data was analyzed for each group. One animal was excluded from the DBS-Pilo group due to the incorrect positioning of the recording electrodes, confirmed later by histology. The percentage distribution of IEDs was found to be similar in the DBS-Pilo and DBS-Control group, as the most frequent IEDs were represented by spikes with a proportion of ~72%, followed by sharp waves (~17%), and polyspikes (~11%). In SHAM-Control rats no polyspikes were detected

**Figure 3:**
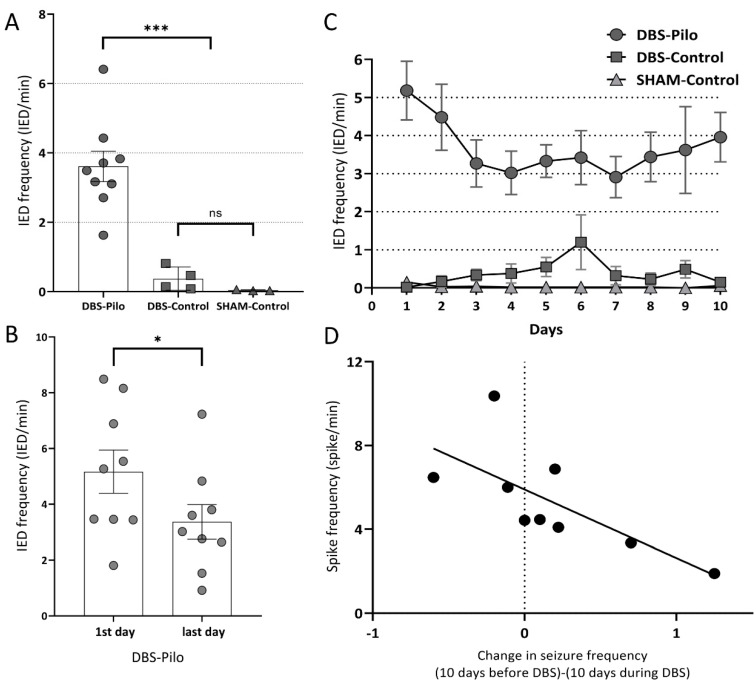
Interictal discharges. (**A**) IED (interictal epileptiform discharge) frequency in different animal groups (dots: individual values, box, and whisker: mean ± SEM). The average IED of DBS-Pilo animals was compared to the controls (*** *p* < 0.001, ns = non-significant). When the two control populations were compared there was no significant difference between them. (**B**) IED frequency of DBS-Pilo animals on the first and the last day of stimulation (dots, box, and whisker as in panel A, * *p* < 0.05). 7 out of the 9 animals showed a decrease. (**C**) IED frequencies during the 10 days of deep brain stimulation. Dots represent the average IED rate on a given day in the given group. A decrease is observed in the pilocarpine group in the first couple of days, with a slight increase in the last days. On the 6th day, the control group had an increase in IED numbers, but this was not permanent. (**D**) Correlation between average spike rate and change in seizure rate of individual animals. There was a significant correlation (r = −0.71, *p* < 0.05). Positive values on the horizontal axis indicate a decrease in seizure frequency while negative values increase.

During the 10-day long electrophysiological recording, the DBS-Pilo group had an average of 3.61 interictal discharges/minute (IED/min), DBS-Control group had 0.37 IED/min while the SHAM-Control 0.04 IED/min. The number of IEDs was significantly higher (*p* < 0.001) in the DBS-Pilo group compared to the control groups, while there was no significant difference between the two control groups (*p* = 0.15) (Figure 3A).

The IED frequency decreased in the DBS-Pilo group during the 10 days of stimulation: on the 10th day, the IED rate was significantly lower by 34.8% compared to the first day (5.17 ± 0.77 vs. 3.37 ± 0.62 IED/min) (Figure 3B). The most evident decrease in the IED rate in the DBS-Pilo group is observed during the first 3 days of DBS, with a slight increase in the last days (Figure 3C). The reduction in seizure rate (the difference between the seizure rate during the 10 days of DBS compared to 10 days before DBS) had a negative correlation with the average IED rate. Subtype analysis of IEDs had shown a negative correlation with spike rate and polyspike rate in individual animals (r = −0.71, *p* < 0.05 and r = −0.69, *p* < 0.05) (Figure 3D). However, there was no correlation between the rate of reduction in seizure numbers and sharp waves. Each animal had more IEDs on one side (left or right hippocampus). The side distribution was not systematic, i.e., there were animals with more IEDs on the left side, and others with more on the right side. During the 10 days of DBS, this IED frequency lateralization did not change significantly.

### 3.3. Spectral Analysis: Theta Power Density

**Figure 4:**
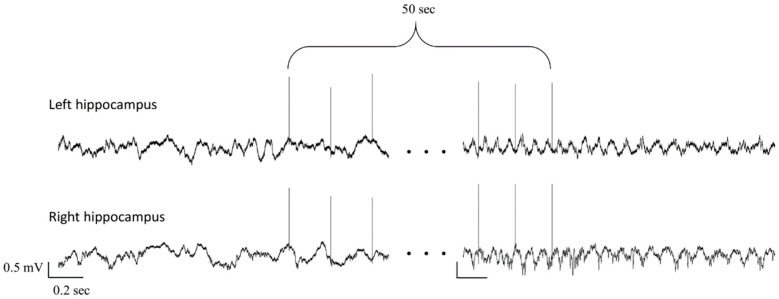
Local field potential (LFP) of the 2 hippocampi before and after a stimulation train. Only the first and the last three stimuli of the train are shown. Note the irregular activity before stimulation, while at the end of the stimulation 5–6 Hz regular LFP can be seen.

**Figure 5:**
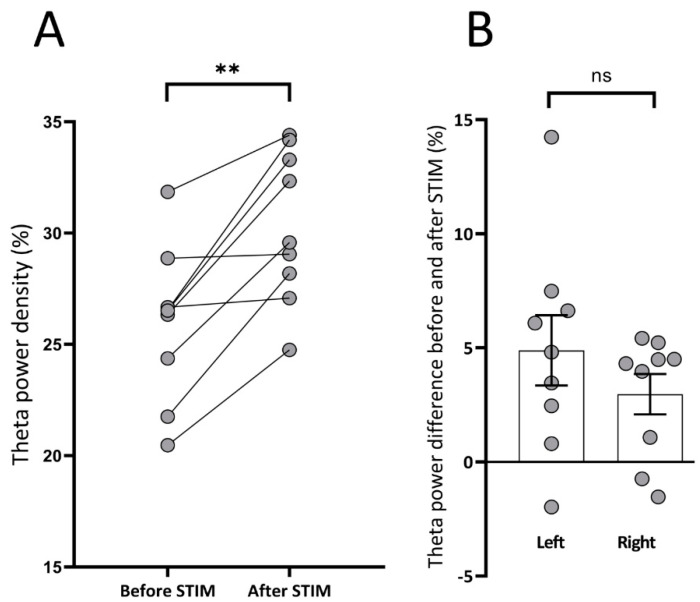
The effect of deep brain stimulation on theta power density. (**A**) The average theta power density of individual animals before and after the stimulation trains. Dots represent the average theta power of an animal. All animals had an increase in theta power following the stimulation train (** *p* < 0.01). (**B**) Theta power density changes in the two hippocampi. Positive values indicate an increase, negative values a decrease in the absolute theta power after stimulation trains in the left and right hippocampus (dots: individual animals, box and whisker: mean ± SEM, ns = non-significant). Two animals had a decrease in theta power on the right side and one animal on the left, but these three were different animals, and the average of left and the right side was also increased in these cases.

The average theta power density 30 s before stimulation was compared to the average theta power density during the 30 s after the last stimulation train. Post-stimulation theta showed an overall amplification of 17% compared to initial hippocampal theta preceding stimulus train (25.9% ± 1.1 vs. 30.3% ± 1.1; *p* < 0.01). This increase was present in all animals if the left and right sides were averaged (Figure 4 and Figure 5A). As stimulation was applied unilaterally at the left side, we considered it important to evaluate whether there was a difference between the two hippocampi regarding theta power increase. Before stimulation, there was no significant difference between the theta power of the left and right sides. Theta power on the left side increased by 19.7% (from 25.7% ± 0.8 vs. 30.7% ± 1.4; *p* < 0.01), while on the right by 10.8% (27.5% ± 1.4 vs. 30.4% ± 1.3; *p* < 0.01). Although the increase was more pronounced on the side of the stimulation (left), there was no significant difference between the change on the left and right sides (Figure 5B). There was no significant difference in theta power increase between subsequent stimulation trains and between different days.

### 3.4. Phase-Amplitude Coupling

**Figure 6:**
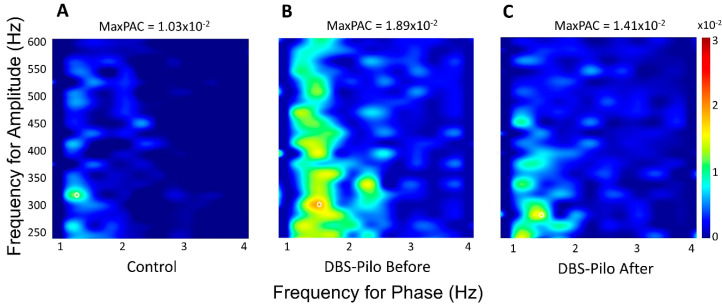
Representative comodulograms of phase-amplitude coupling (PAC) of individual animals showing the PAC strength between delta and fast-ripple frequency pairs. The coupling strength is color-coded (scale bar on the right). (**A**) The PAC comodulogram of the control animal is from a SHAM-Control rat. (**B**) The epileptic animals exhibited a stronger MaxPAC (white circle) which (**C**) was reduced after deep brain stimulation.

**Figure 7:**
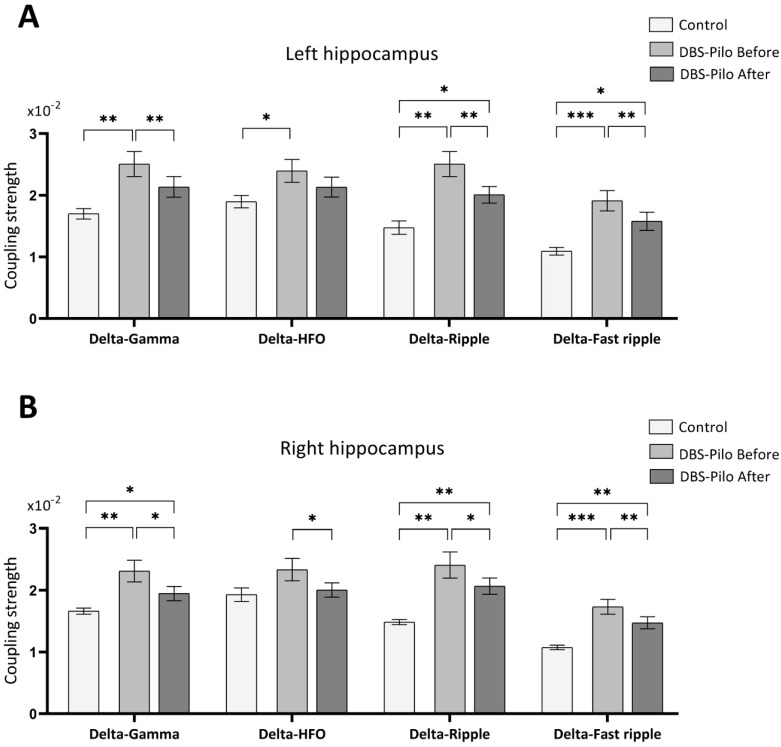
Maximum coupling strength between hippocampal delta and higher frequencies. All values are the average of 10 days. (**A**) Comparison of the coupling strength of control groups (SHAM-Control and DBS-Control together), DBS-Pilo Before and DBS-Pilo After in the left hippocampus. and (**B**) in the right hippocampus. Note that epileptic animals have significantly higher PAC values than controls. There is a global decrease after deep brain stimulation in PAC strength and every decrease is significant, except the delta-HFO PAC in the left hippocampus. Data were represented as mean ± SEM, * *p* < 0.05; ** *p* < 0.01; *** *p* < 0.001.

Coupling strength was evaluated on the hippocampal recordings on both sides. First, to see if PAC was modified in epileptic rats, the 10-day average of pre-stimulation periods of epileptic animals and control animals were compared. The coupling strength was higher in the epileptic rats in all studied frequency bands (delta-gamma, delta-HFO, delta-ripple, and delta-fast ripple, as defined in Methods), on both sides. All differences were significant except the delta-HFO PAC on the right hippocampal recording (Table 1; Figure 6 and Figure 7). There was no significant difference between the two hemispheres. To test the effect of DBS on PAC the pre-stimulation values were compared with the PAC values measured in the 5-min periods following the stimulation trains. We found a significant decrease in every frequency band of the f(A) in the DBS-Pilo animals, except the delta-HFO coupling in the left hippocampus (Table 1; Figure 6 and Figure 7). Again, there was no significant difference between the right and left hippocampus. In the majority of frequency bands (except the left delta-gamma) the decreased PAC values after DBS still differed significantly from those measured in controls. The PAC strength did not change in the DBS-Control group after the stimulation trains, and there was no difference between the SHAM-Control and DBS-Control groups. Therefore, the PAC values of the control animals were pooled and represent the first 5 min periods of daily recordings from the SHAM-Control and DBS-Control groups.

PAC between delta and higher frequencies (gamma, ripple, and fast ripple) measured in the left hippocampus of the epileptic rats had a negative correlation with the change in daily seizure rate during the 10 days of DBS compared to 10 days before it (Table 2). Those animals that had fewer seizures during the 10 days of DBS than before it, had weaker phase-amplitude coupling before and after the stimulation trains too. No significant differences in the DBS-Pilo After PAC values were measured between different stimulation days.

## 4. Discussion

The present study proposed to test the effects of amygdala LFS in the pilocarpine model of TLE in rats, and more importantly, to find out possible mechanisms of DBS by studying the electrophysiological properties of the hippocampus. We found that epileptic animals had significantly higher PAC values and IED rate than controls. The amygdala LFS reduced this pathologically increased phase-amplitude coupling in all epileptic animals and reduced seizures more effectively in animals having fewer IEDs during DBS, while it did not affect the PAC in healthy animals. The average theta power, which was suggested to be reduced during epileptogenesis [50,51,52], was increased in the 30 s periods following the stimulation trains. All these phenomena could have a role in reducing the pathologically increased synchrony observed in epilepsy.

We observed that regular low-frequency stimulation with 4 Hz of the left BLA could significantly reduce the seizure duration. Nonetheless, its efficacy in reducing seizures rate remained limited, as only in 2/3 of the animals was the seizure rate reduced. We hypothesize that short stimulation periods might explain the modest effect on the seizure rate. The effect of DBS is highly dependent on stimulation parameters, like the number and duration of stimulus epochs [53] or the co-application of anticonvulsant drugs [54]. Stimulation frequency is also a very important parameter, as high-frequency stimulation (HFS; >50 Hz) and low-frequency stimulations (LFS; <5 Hz) can both reduce seizure numbers in humans and rodents, but intermediary frequencies cannot [55,56,57]. In humans, the most widely used antiepileptic DBS protocol consists of stimulating the thalamus with high frequency (e.g., 130 Hz, with an “on for one minute, off for five minutes” protocol [21]). It has been observed a long time ago that stimulation of the amygdala with intermediary frequency can elicit hippocampal epileptiform activity (known as kindling), and is a widely used model to induce epilepsy in rodents [58]. Contrarily, LFS of the amygdala from 1 to 4 Hz can prevent or reduce the pathological electrical activity caused by the electrical kindling of the amygdala, but at least four packages of 100–200 pulses must be administered daily for antiepileptic effect [19,20,59]. Temporally unstructured (nonperiodic) stimulation with an average frequency of 4 Hz was reported to be more effective in reducing the PTZ induced seizure threshold than regular interpulse interval stimulation [26,28]. The same group reported that pilocarpine-induced seizure rate and duration were reduced significantly in the chronic phase by nonperiodic LFS of the right amygdala while they found that regular (periodic) stimulation had no significant effect [25]. However, they only analyzed a shorter, 4 day period, 6 h daily, and the stimulation was done continuously during these 6 h. In our study, the stimulation was applied to the left BLA for a longer period (10 days), but it lasted only 30 min/day. According to the literature, using bilateral stimulation was superior to unilateral, but unilateral stimulation of the left or right amygdala was proved to be effective regardless of the seizure onset side. The right amygdala was used as a target in more studies, but when the sides were compared, stimulation of the left was superior and had better seizure suppressing effects than the right [20,25,26,60].

The pathological electrophysiological activity caused by the altered synchronicity and network oscillations during epileptogenesis [61] is very similar in the pilocarpine treated animals and human TLE patients [44]. Interictal epileptiform discharges (IED), e.g., spikes, polyspikes, sharp waves, and slow-sharp waves are signs of transient pathological hyper-synchronization of neuronal networks [34]. Here we show their potential role as a marker of seizure outcome in rats treated by DBS: the lower the average IED rate, spike rate, and polyspike rate was during the treatment period, the more suppress of seizures could be obtained by electrical stimulation. However, there was no correlation between the rate of reduction in seizure numbers and sharp waves and slow-sharp waves, a finding which reveals that the different types of IEDs have contrasting relation with the clinical state and they must be evaluated separately. Still, by counting IED numbers one simply cannot assess seizure frequency but can detect the presence of the disease as IED numbers in the epileptic group were significantly higher than in seizure-free animals. We did not observe any immediate effect of stimulation on IED frequency, as it remained unchanged during the stimulation packages and the period immediately following them. In contrast, by the 3^rd^ day of treatment IED numbers dropped, and on the 10^th^ day of stimulation, the IED rate was significantly lower than at the beginning of the stimulation. The asymmetric side distribution (constantly more interictal discharges appeared on the left or right hippocampus) was not modified by the unilateral stimulation. We confirmed that unilateral stimulation modulated both hemispheres (a phenomenon observed by the theta power and PAC analysis too), which is consistent with the literature data [26,60,62]. 

As their number is usually higher in epileptic patients [63,64], IEDs have been recognized as useful epileptic markers, although they could exhibit both pro- and anti-seizure effects and their numbers do not necessarily correlate with seizure frequency [34,65]. Similar to our findings reduced interictal activity was observed in humans treated by LFS [24,66,67]. Moreover, a recently published 7 year follow-up study had shown that the interictal spike rate could be related to the average seizure number in epileptic patients treated by DBS, and can differentiate patients with good clinical outcome [68].

The modulation of hippocampal rhythms may play an important role in TLE treatment. Here we reflect on the implication of one particular hippocampal rhythm, the theta rhythm, in temporal lobe epilepsy, and demonstrate its role in the given therapeutic paradigm. In this study we demonstrate that amygdala LFS can amplify theta-band power, offering a possible explanation for the antiepileptic effect of amygdala deep brain stimulation in TLE. As theta power increase was not present after 24 h, we may assume that the effect is present only in the short-term, and probably continuous or responsive stimulation could be more efficient by keeping the theta power constantly increased or increasing whenever is necessary. The rate of increase was higher on the side of the stimulation electrode, but the difference was not significant between the two sides, thus probably the unilateral stimulation can affect both sides. Similar to Bettus et al., we found that theta power changes were not correlated with the frequency of interictal spikes [35]. As the amygdala and hippocampus are strongly interconnected [69] it is expected that the amygdala LFS could facilitate long-term depression in the hippocampus [70], and may modulate the network synchronicity in the limbic system [71]. It was reported by others that physiological theta-power decreased during the interictal periods of human TLE patients and in kainate and pilocarpine rat models of TLE [35,50,51,52,72,73]. In-vitro experiments had shown that diazepam (an antiepileptic drug) significantly increases the theta power [74]. Although the associated mechanisms are not clear, it was proposed that the loss of dendrite inhibiting CA1 interneurons could lead to decreased theta rhythm [75]. There is an increasing amount of evidence that during a spontaneous or induced increase of the theta power (so far by electrical stimulation of the septum, microinjection with carbachol or tail pinch) can suppress epileptiform activity and seizures, increase seizure threshold and improve cognitive performance in epileptic rats [73,76,77].

Phase-amplitude coupling (PAC) proved useful to investigate local and large-scale communications and synchronism amongst cell ensembles [37,78,79,80]. Here we show that PAC strength is increased in pilocarpine treated animals compared to healthy ones, in the early chronic phase of the disease. The difference was most outstanding in the delta-fast ripple coupling, which highlights the relevance of fast ripples as nowadays they acquired a critical role in the diagnosis of epilepsy [81]. We considered it important to set the upper limit of the fast ripple band to 600 Hz since fast ripples are reportedly present in the rat hippocampus between 400 Hz–600 Hz too [82]. By using distinct bands for modulated frequencies, we aimed to determine whether there is a modulated oscillation which indicates with relatively higher sensitivity the presence of pathological coupling. There are complex computational models to study the mechanisms of generation of PAC. These models over time could be used to understand the mechanisms of different interventions on PAC [83].

PAC was reportedly stronger in epileptic humans and rodents during pre-ictal and ictal periods and in the seizure onset zones [38,39,84]. In frontal cortex slice preparations, cross-frequency coupling between theta and gamma was increased when excitatory chemicals, like kainate were applied [74]. Moreover, two recent studies reported increased PAC during interictal periods in pilocarpine treated rats. One found increased tPAC (the same parameter we used) compared to control animals during the latent phase (the two weeks following SE) between delta and higher frequency bands (from 20 to 250 Hz) in the hippocampus. The 20–250 Hz frequency band was not analyzed separately by them, but their absolute tPAC values are similar to those found by our group [85]. The other study found stronger coupling 5 weeks after SE in the CA1 region, between delta and all analyzed higher frequencies. They separated higher frequencies similar to us, with the higher limit of fast ripple being set to 400 Hz, while in our case was set to 600 Hz. Other brain regions didn’t show increased coupling. They measured coupling by using the modulation index parameter, therefore our values cannot be compared directly with their tPAC values [86]. Our results expand these observations, showing elevated PAC strength approximately 3 months after SE. This shows that altered coupling persists from the early chronic period of TLE.

Recently it was proposed to use neuromodulation techniques based on monitoring the phase-amplitude coupling in various neurologic disorders [40], but so far, to our knowledge, there is no information about the effect of electrical stimulation on PAC in epilepsy. Importantly, our results demonstrate for the first time that amygdala LFS can decrease significantly the pathological coupling between delta and higher frequencies, i.e., suggesting that DBS of the brain could exhibit an antiepileptic effect by reducing the affected connectivity strength that emerged from pathologically rearranged network activity [61,87]. However, we should remark that the PAC values were not decreased by DBS to the values measured in healthy controls. Both the difference between epileptic and control animals and the decrease in coupling strength in epileptic animals observed after the stimulation was unequivocal in most of the frequency bands except for the delta-HFO coupling, which suggests that higher frequency bands should be evaluated separately. Similar to Izadi et al., who reported the lack of effect of DBS on theta power in healthy controls, we found that DBS didn’t modify PAC strength in control animals.

We consider, that the antiepileptic effectiveness of DBS could be estimated not only with IEDs but with PAC too; the less strongly were gamma, ripple, and fast ripple bands (obtained from the stimulated hemisphere) coupled with delta rhythm, the more outstanding was the reduction in seizure rate during the treatment period.

These findings should be interpreted carefully due to the short duration of DBS. Further experiments are needed using larger study populations and longer DBS periods and different DBS protocols as well as different epilepsy models. The slight increase in IED frequency in the 10th day suggests that the evaluation of even longer periods may hold valuable information. We attribute the limited effect of DBS on seizure frequency to the short duration of daily stimuli. Histological studies, like Amorim et al. [88], should be made to address the effect of DBS at cellular level and to find possible correlations with the electrophysiological markers presented in our study.

## 5. Conclusions

In the pilocarpine model of temporal lobe epilepsy, average theta power is decreased while the coupling between the delta band phase and higher frequencies amplitude is increased during the chronic phase of the disease. Amygdala LFS reduced mean seizure duration, interictal epileptiform discharge, and seizure rate in most of the animals. The electrical stimulation increased the theta power and reduced the pathologically increased phase-amplitude coupling in epileptic rats, possible mechanisms by which it may exhibit the antiepileptic effect. LFS was not affecting the PAC values in healthy rats, it only affected epileptic animals. Measuring PAC is a novel and promising tool to characterize the large-scale electrical activity and to monitor the therapeutic effect of DBS in epilepsy. Moreover, interictal spike rate and PAC analysis can be a useful tool for diagnosis of the disease and to predict individuals with good response to therapeutic electrical stimulation.

## Figures and Tables

**Table 1:** Maximum PAC between delta (1–4 Hz) and fast (30–600 Hz) oscillations.

Electrode Position	Group	Delta-Gamma	Delta-HFO	Delta-Ripple	Delta-Fast Ripple
L.H.	Control	1.70 × 10^−2^ ± 9 × 10^−4^	1.9 × 10^−2^ ± 1 × 10^−3^	1.47 × 10^−2^ ± 1.1 × 10^−3^	1.09 × 10^−2^ ± 6 × 10^−4^
	DBS-Pilo Before	2.51 × 10^−2^ ± 2 × 10^−3^	2.4 × 10^−2^ ± 1.8 × 10^−3^	2.51 × 10^−2^ ± 2 × 10^−3^	1.91 × 10^−2^ ± 1.7 × 10^−3^
	DBS-Pilo After	2.14 × 10^−2^ ± 1.7 × 10^−3^	2.13 × 10^−2^ ± 1.6 × 10^−3^	2.01 × 10^−2^ ± 1.4 × 10^−3^	1.58 × 10^−2^ ± 1.5 × 10^−3^
R.H.	Control	1.66 × 10^−2^ ± 5 × 10^−4^	1.93 × 10^−2^ ± 1.1 × 10^−3^	1.48 × 10^−2^ ± 4 × 10^−4^	1.12 × 10^−2^ ± 5 × 10^−4^
	DBS-Pilo Before	2.31 × 10^−2^ ± 1.8 × 10^−3^	2.33 × 10^−2^ ± 1.8 × 10^−3^	2.41 × 10^−2^ ± 2.1 × 10^−3^	1.73 × 10^−2^ ± 1.2 × 10^−3^
	DBS-Pilo After	1.95 × 10^−2^ ± 1.2 × 10^−3^	2 × 10^−2^ ± 1.2 × 10^−3^	2.07 × 10^−2^ ± 1.3 × 10^−3^	1.47 × 10^−2^ ± 1 × 10^−3^

Control: Phase-amplitude coupling (PAC) values of the control animals during the first 5-min periods (SHAM-Control and DBS-Control groups) of daily recordings; DBS-Pilo Before: PAC values of DBS-Pilo group during the 5-min periods before the stimulation trains; DBS-Pilo After: PAC values of the DBS-Pilo group during the 5-min periods after the stimulation trains. All values are the averages of the 10-day recordings. L.H.: left hippocampus; R.H.: right hippocampus.

**Table 2:** Correlation between change in seizure rate and PAC strength.

Time Interval	Electrode Position	Pearson Test	Delta-Gamma	Delta-HFO	Delta-Ripple	Delta-Fast Ripple
Before DBS	Left hippocampus	r	−0.86	−0.63	−0.86	−0.75
		p	0.0027 **	0.0713	0.0031 **	0.0201 *
	Right hippocampus	r	0.003	−0.48	−0.65	−0.62
		p	0.9940	0.1872	0.0570	0.0761
After DBS	Left hippocampus	r	−0.77	−0.67	−0.84	−0.88
		p	0.0143 **	0.0487 *	0.0048 **	0.0017 **
	Right hippocampus	r	−0.03	−0.36	−0.45	−0.73
		p	0.9431	0.3448	0.2226	0.0251 *

* *p* < 0.05; ** *p* < 0.01.

## Supplementary Materials

The following are available online at https://www.mdpi.com/2076-3425/10/11/856/s1.

## Abbreviations

BLA	Basolateral Amygdala
DBS	Deep Brain Stimulation
EEG	Electroencephalography
GABA	Gamma-Aminobutyric Acid
HFO	High-Frequency Oscillation
HFS	High-Frequency Stimulation
IED	Interictal Epileptiform Discharge
LFP	Local Field Potential
LFS	Low-Frequency Stimulation
PAC	Phase-Amplitude Coupling
Pilo	Pilocarpine
PTZ	Pentylenetetrazole
SD	Standard Deviation
SE	Status Epilepticus
SEM	Standard Error of the Mean
SRSs	Spontaneous Recurrent Seizures
tPAC	Time-Resolved Phase-Amplitude Coupling
TLE	Temporal Lobe Epilepsy
