# Supplementary material for: Amygdala Low-Frequency Stimulation Reduces Pathological Phase-Amplitude Coupling in the Pilocarpine Model of Epilepsy
Source: Brain Sci. 2020 Nov 13;10(11):856. doi: 10.3390/brainsci10110856 (PMC7696538; doi:10.3390/brainsci10110856)
Supplement: Supplementary file 1 [file brainsci-10-00856-s001.pdf]

Supplementary file for the Manuscript entitled:

Amygdala low-frequency stimulation reduces pathological phase-amplitude coupling in the pilocarpine model of epilepsy

By: István Mihály [1,*], Károly Orbán-Kis [1], Zsolt Gáll [2], Ádám-József Berki [1], Réka-Barbara Bod [1] and Tibor Szilágyi [1]

[1] Department of Physiology, Faculty of Medicine, George Emil Palade University of Medicine, Pharmacy, Science, and Technology of Târgu Mureș, 540142 Târgu Mureș, Romania; karoly.orban-kis@umfst.ro (K.O.-K.); berki.adam@yahoo.com (A.-J.B.); bod.reka@yahoo.com (R.-B.B.), tibor.szilagyi@umfst.ro (T.S.)

[2] Department of Pharmacology and Clinical Pharmacy, George Emil Palade University of Medicine, Pharmacy, Science, and Technology of Târgu Mureș, 540142 Târgu Mureș, Romania; zsolt.gall@umfst.ro (Z.G.);

* Correspondence: istvan.mihaly@umfst.ro; Tel.: +40749768257.

Technical limitations and further technical details of the study

Section 1 – Video recordings and seizure detection

Based on video recordings, only Racine 3-6 stage seizures could be identified. Videos were analyzed offline by manually going through the whole recording with 4x-6x speed. Observers were not blind to the experiments and they had to detect spontaneous behavioral seizures and behavioral abnormalities. As unusual behavior was found, the sequence was re-watched with normal speed, the event's exact start, as well as duration, was noted. If it was a seizure then it was graded according to the revised Racine scale. Observers were not blind to the experimental conditions, but were blind regarding the electrophysiological data, exact timing of the stimulus, occurrence of IEDs, etc. Concomitant EEG was recorded, only during the stimulation periods and the corresponding pre- and post-stimulation intervals. Video-EEG would improve seizure detection as R1-R2 seizures cannot be identified without EEG recording, but the high majority of seizures in the post-status epilepticus models are R3 or higher-grade seizures and R3-R6 seizures can be reliably identified based on video recordings only [1][2]. Also, de Oliveira et al. [3] showed that electrical stimulation of the amygdala suppresses behavioral seizures. Based on these data from the literature and in accordance with our working hypothesis we tried to shine light on the possible effects of DBS on the electrical activity and the main focus was not on the effect of seizure pattern. In this regard, our approach was similar to several recent studies [4].

Section 2. – Stimulation artifacts on EEG

Stimulation artifacts on the EEG could not be avoided. We chose not to filter out the stimulation artifacts as any filter could distort the phase of the oscillations. Interestingly De Hemptinne et al. [5] found that stimulation artifacts may not alter the result of PAC analyses. Nevertheless, the parameters which could have been affected by the stimulation artifact (PAC and spectral power density) were not measured and analyzed while the stimulation burst was on, only during the five-minute periods before or immediately following them. IEDs were measured along the entire length of the 30-minute long recordings (during the stimulation bursts as well). However, their identification was not limited by the stimulation artifacts, as the duration of the stimulation artifact is very short compared to the IEDs (>30 ms).

Further technical details

Section 3. - Electrode positions

The distance of the stimulation electrode (always left side) to the left hippocampal recording electrode was 5.4 mm, while to the right hippocampal recording electrode it was 9 mm. The distance of the stimulation electrode (always left side) to the left hippocampal recording electrode was 5.4 mm, while to the right hippocampal recording electrode it was 9 mm. The stimulation artifact propagates by volume transmission through the brain parenchyma and it is recorded by the electrodes in the hippocampus, but it is far too weak to have a significant direct effect on hippocampal neurons. Nevertheless, stimulation of the amygdala modulates the hippocampal activity through its direct and indirect neural connections, as these two areas are strongly interconnected [6].

References to Supplementary File

[1] J. Pitsch, A. J. Becker, S. Schoch, J. A. Müller, M. de Curtis, and V. Gnatkovsky, "Circadian clustering of spontaneous epileptic seizures emerges after pilocarpine-induced status epilepticus," *Epilepsia*, vol. 58, no. 7, pp. 1159–1171, 2017.

[2] R. Bajorat, M. Wilde, T. Sellmann, T. Kirschstein, and R. Köhling, "Seizure frequency in pilocarpine-treated rats is independent of circadian rhythm.," *Epilepsia*, vol. 52, no. 9, pp. e118-22, 2011.

[3] J. C. de Oliveira, D. de C. Medeiros, G. H. de Souza e Rezende, M. F. D. Moraes, and V. R. Cota, "Temporally unstructured electrical stimulation to the amygdala suppresses behavioral chronic seizures of the pilocarpine animal model," *Epilepsy Behav.*, vol. 36, pp. 159–164, 2014.

[4] M. Vrinda, A. Sasidharan, S. Aparna, B. N. Srikumar, B. M. Kutty, and B. S. Shankaranarayana Rao, "Enriched environment attenuates behavioral seizures and depression in chronic temporal lobe epilepsy," *Epilepsia*, vol. 58, no. 7, pp. 1148–1158, 2017.

[5] C. De Hemptinne, N. C. Swann, J. L. Ostrem, E. S. Ryapolova-Webb, *et al.*, "Therapeutic deep brain stimulation reduces cortical phase-amplitude coupling in Parkinson's disease," *Nat. Neurosci.*, vol. 18, no. 5, pp. 779–786, 2015.

[6] A. Pitkänen, M. Pikkarainen, N. Nurminen, and A. Ylinen, "Reciprocal connections between the amygdala and the hippocampal formation, perirhinal cortex, and postrhinal cortex in rat. A review.," *Ann. N. Y. Acad. Sci.*, vol. 911, pp. 369–391, 2000.
